# Supplementary figures and images for: The Role of Genetically Distinct Central Amygdala Neurons in Appetitive and Aversive Responding Assayed with a Novel Dual Valence Operant Conditioning Paradigm
Source: eNeuro. 2023 Sep 4;10(9):ENEURO.0319-22.2023. doi: 10.1523/ENEURO.0319-22.2023 (PMC10488222; doi:10.1523/ENEURO.0319-22.2023)

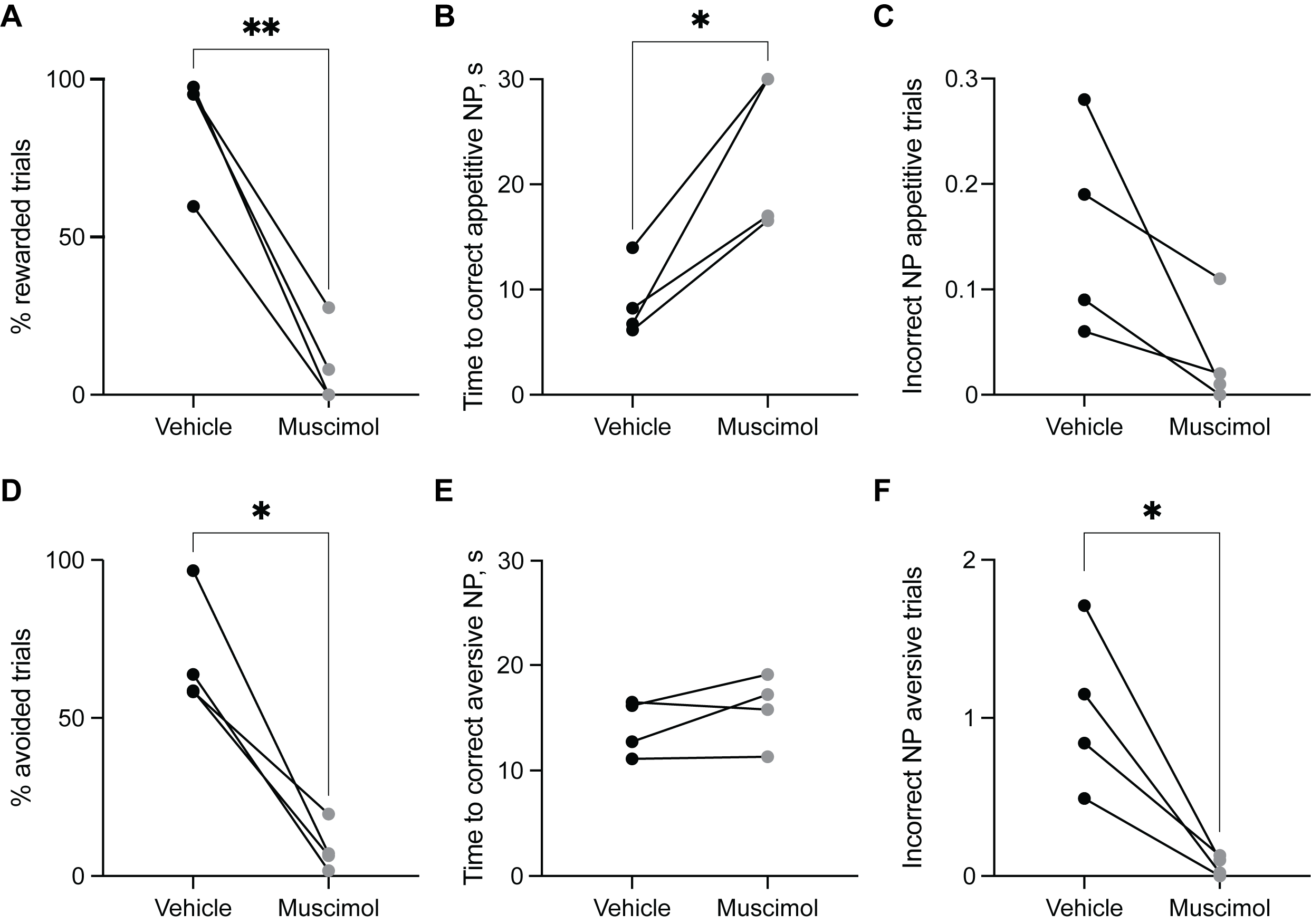

Supplement: Extended Data Figure 2-1 — Dual valence task performance requires the CeA. Mice were implanted with bilateral cannulae targeting the CeA and muscimol (400 ng/side) or vehicle was infused prior to testing. A, Muscimol treatment significantly impaired appetitive operant performance. B, Muscimol treatment significantly increased the latency to correct response on appetitive trials. Two mice did not respond on any appetitive trials, so latency was capped at the trial duration (30 s). C, Muscimol caused a nonsignificant decrease in the average number of incorrect responses during appetitive trials. D, Muscimol treatment significantly impaired operant performance on avoidance trials. E, The latency to correct response on aversive trials was not affected by muscimol. F, Muscimol caused a nonsignificant decrease in the average number of incorrect responses during aversive trials. Download Figure 2-1, TIF file. [file enu-eN-MNT-0319-22-s01.tif]

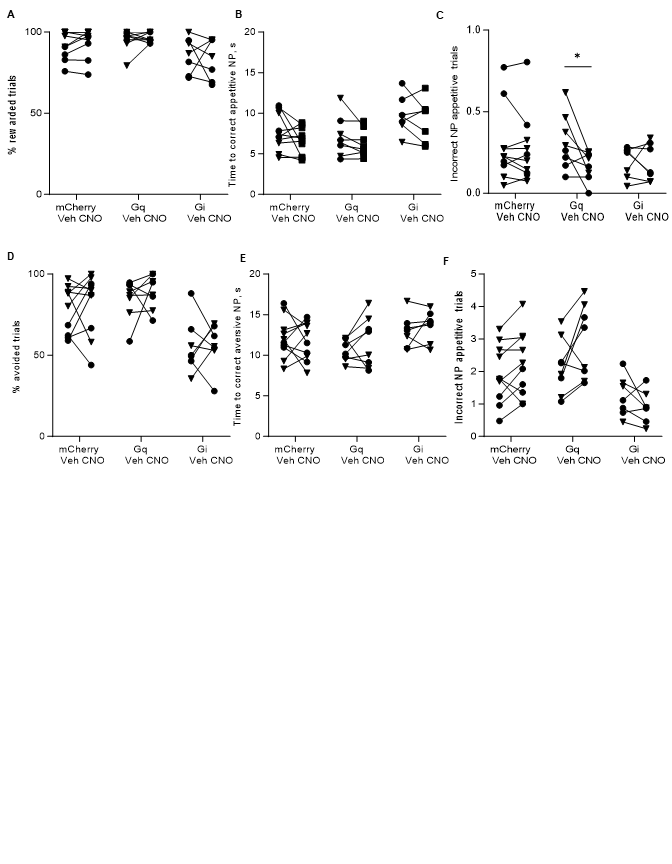

Supplement: Extended Data Figure 4-1 — Vehicle and CNO data for the SOM-Cre chemogenetic groups. A, There were no significant differences between vehicle and CNO treatments on the percentage of rewarded trials. B, There were no significant treatment effects on the latency to correct response on appetitive trials. C, In the Gq group, CNO treatment caused a significant reduction in the number of incorrect responses during appetitive trials (paired t test, t(7) = 2.5, p = 0.04). D, There were no significant differences between vehicle and CNO treatments on the percentage of correct avoidance trials. E, There were no significant effects of CNO on the latency to correct avoidance response. F, There were no significant effects of CNO on the number of incorrect responses during aversive trials. *p < 0.05. Triangle symbols = males, circles = females. Download Figure 4-1, TIF file. [file enu-eN-MNT-0319-22-s02.tif]

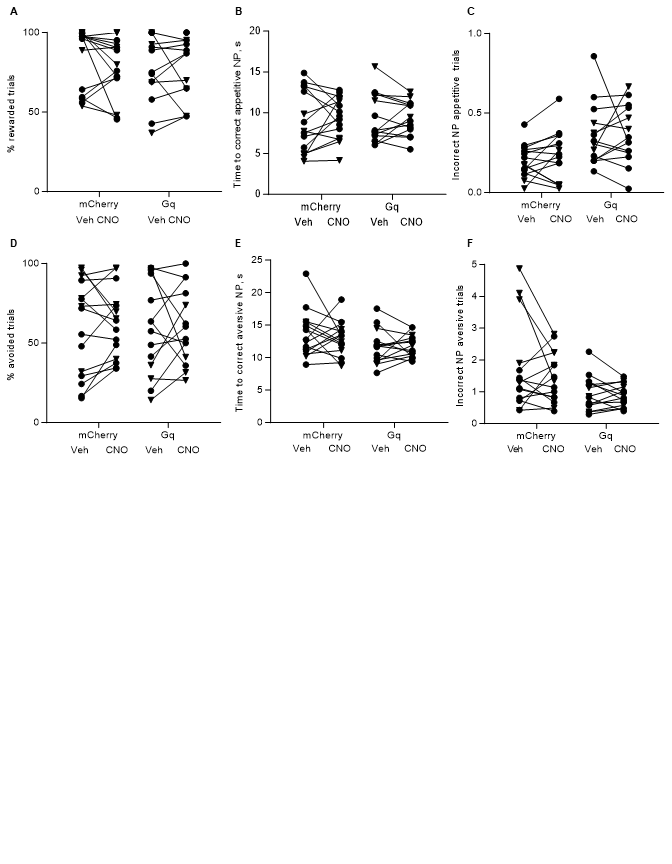

Supplement: Extended Data Figure 5-1 — Vehicle and CNO data for the CRF-Cre chemogenetic groups. There were no significant differences between vehicle and CNO treatments on (A) the percent of rewarded appetitive trials; (B) the latency to correct response on appetitive trials; (C) the number of incorrect responses during appetitive trials; (D) percent avoidance; (E) the latency to correct avoidance response; (F) the number of incorrect responses during aversive trials. Download Figure 5-1, TIF file. [file enu-eN-MNT-0319-22-s03.tif]

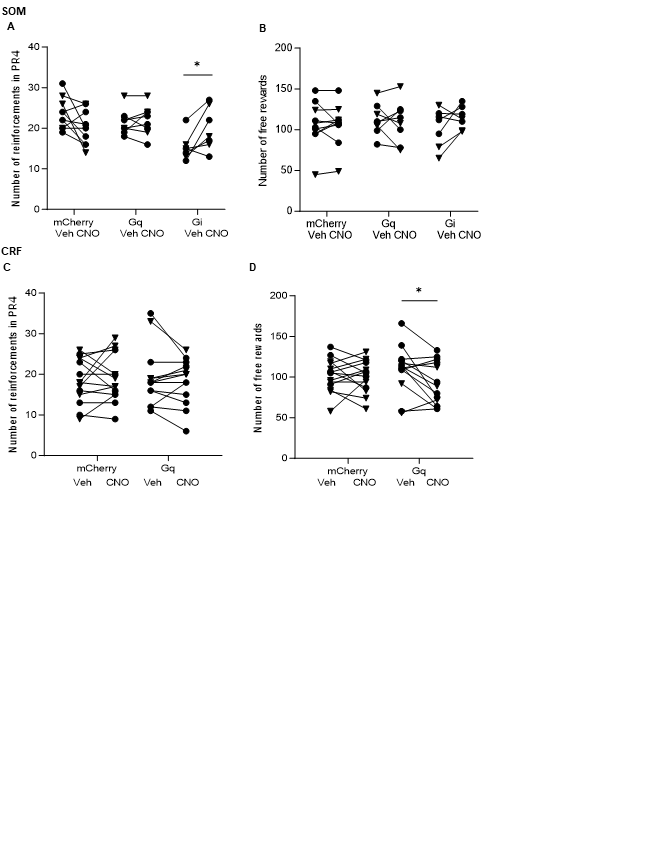

Supplement: Extended Data Figure 6-1 — Vehicle and CNO data for the appetitive motivation and free reward consumption tests. A, CNO induced a significant elevation in the number of reinforcements during the progressive ratio test in the inhibitory DREADD group (paired t test, t(6) = 2.7, p = 0.03). B, There was no significant effect of CNO on free reward consumption in the SOM+ groups. C, There was no significant effect of CNO on appetitive motivation in the CRF+ groups. D, CNO reduced free reward consumption in the excitatory DREADD CRF group (paired t test, t(12) = 2.4, p = 0.03). *p < 0.05. Triangle symbols = males, circles = females. Download Figure 6-1, TIF file. [file enu-eN-MNT-0319-22-s04.tif]
